# Dengue and COVID-19: two sides of the same coin

**DOI:** 10.1186/s12929-022-00833-y

**Published:** 2022-07-03

**Authors:** Gathsaurie Neelika Malavige, Chandima Jeewandara, Graham S. Ogg

**Affiliations:** 1 Allergy Immunology and Cell Biology Unit, Department of Immunology and Molecular Medicine, Faculty of Medical Sciences, University of Sri Jayewardenepura, Nugegoda, Sri Lanka; 2 MRC Human Immunology Unit, MRC Weatherall Institute of Molecular Medicine, University of Oxford, Oxford, UK

**Keywords:** Dengue, SARS-CoV-2, COVID-19, Severe illness, Immunopathology, Innate immunity, Antibodies, T cells, Endothelial dysfunction

## Abstract

**Background:**

Many countries in Asia and Latin America are currently facing a double burden of outbreaks due to dengue and COVID-19. Here we discuss the similarities and differences between the two infections so that lessons learnt so far from studying both infections will be helpful in further understanding their immunopathogenesis and to develop therapeutic interventions.

**Main body:**

Although the entry routes of the SARS-CoV-2 and the dengue virus (DENV) are different, both infections result in a systemic infection, with some similar clinical presentations such as fever, headache, myalgia and gastrointestinal symptoms. However, while dengue is usually associated with a tendency to bleed, development of micro and macrothrombi is a hallmark of severe COVID-19. Apart from the initial similarities in the clinical presentation, there are further similarities between such as risk factors for development of severe illness, cytokine storms, endothelial dysfunction and multi-organ failure. Both infections are characterised by a delayed and impaired type I IFN response and a proinflammatory immune response. Furthermore, while high levels of potent neutralising antibodies are associated with protection, poorly neutralising and cross-reactive antibodies have been proposed to lead to immunopathology by different mechanisms, associated with an exaggerated plasmablast response. The virus specific T cell responses are also shown to be delayed in those who develop severe illness, while varying degrees of endothelial dysfunction leads to increased vascular permeability and coagulation abnormalities.

**Conclusion:**

While there are many similarities between dengue and SARS-CoV-2 infection, there are also key differences especially in long-term disease sequelae. Therefore, it would be important to study the parallels between the immunopathogenesis of both infections for development of more effective vaccines and therapeutic interventions.

## Background

Although SARS-CoV-2 is reported to have infected over 500 million individuals with at least 6.2 million individuals succumbing to COVID-19 by April 2022 [90], the true direct and indirect death toll due to COVID-19 is estimated to be much higher [126]. Despite the availability of several safe and effective vaccines for COVID-19, the emergence of SARS-CoV-2 variants that evade immunity has posed challenges in controlling outbreaks [33]. Due to the unprecedented cooperation between scientists, sharing of data and availability of funding, by early 2022 ten vaccines had received emergency use licensing by the WHO for the prevention of COVID-19 [128]. This contrasts with many other neglected tropical infections such as dengue, despite causing deaths in 0.53 per 100,000 population in 2017 [139].

Although there are reports of epidemics caused by infection with the dengue virus (DENV) in the 1780s, epidemics resulting in dengue haemorrhagic fever (DHF) or dengue shock syndrome (DSS), which are severe forms of dengue infection, was initially reported in the 1950s in South East Asia [69]. However, dengue infections have gradually increased over time due to many factors such as climate change resulting in increase in temperatures, urbanization, increase mobility and overcrowding [108]. Although there is no specific treatment for dengue, intense monitoring to detect vascular leak and other supportive management has reduced case fatality rates (CFRs) to < 0.3% in most countries [19, 83], although in some countries such as in India the CFRs are estimated to be 2.6% [95]. The CFRs in patients with severe dengue was shown to be around 5.9% for younger children while it was as high as 32.6% in patients ≥ 60 years of age in Brazil [79]. Therefore, it is evident that dengue is an important cause of morbidity and mortality in countries in the tropical and subtropical regions.

While SARS-CoV-2 infects individuals via the respiratory route, the DENV infects individuals following a bite of an infected mosquito of the Aedes species. However, it is well established that COVID-19 is not a mere respiratory infection but is a systemic illness. Furthermore, many of the initial clinical symptoms such as fever, myalgia, joint pain, headache, lethargy, abdominal pain, diarrhoea, vomiting and sometimes sore throat is frequently seen in both infections, making it difficult to clinically differentiate dengue from COVID-19 during early illness [86, 129]. Therefore, many countries that experienced regular outbreaks due to dengue are now faced with the double burden of dengue and COVID-19 [47, 52]. Apart from the initial similarities in the clinical presentation, there are many similarities between these two infections such as certain risk factors for severe illness, immunopathogenesis, antibody and T cell responses, cytokine storms, endothelial dysfunction and multi-organ failure. However, there are certain differences such as haemorrhage in dengue compared to thrombosis occurring in COVID-19. In this review, we discuss the similarities and differences between the two infections so that lessons learnt so far from studying both infections will be helpful in further understanding their immunopathogenesis and to develop therapeutic targets.

### Risk factors for severe illness in dengue and COVID-19

**Fig. 1:**
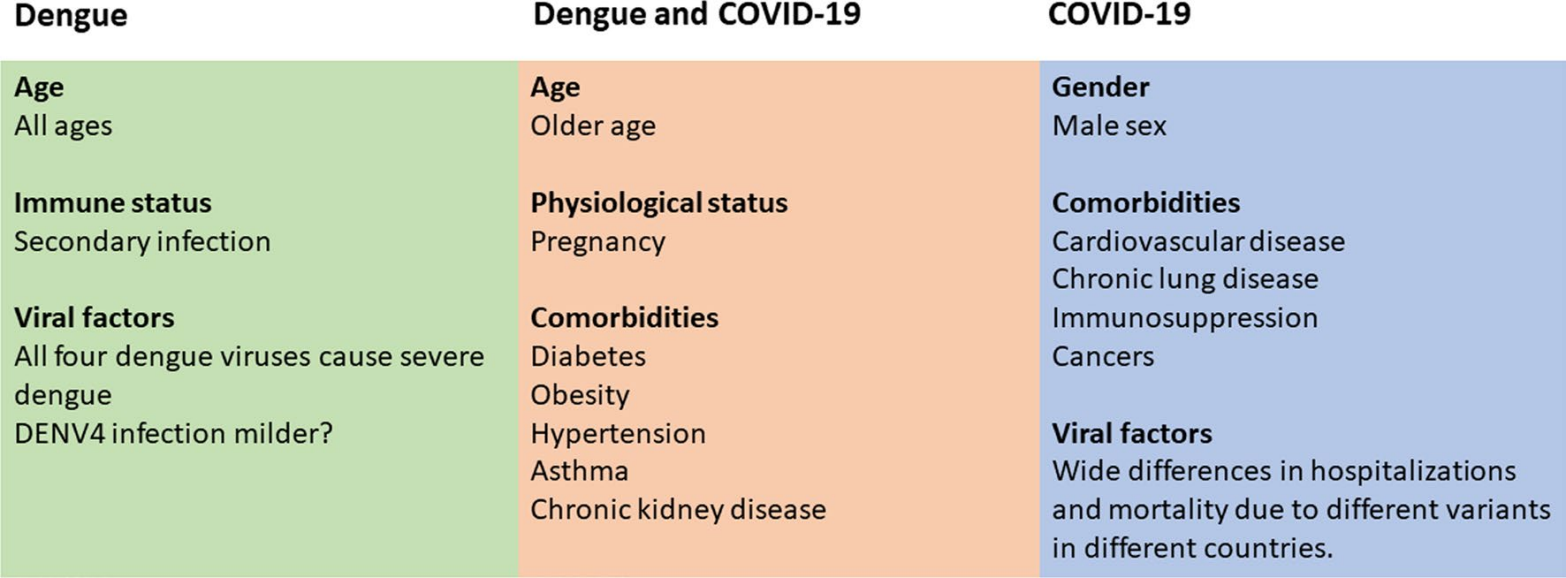
Risk factors for severe dengue and COVID-19. The common changes are highlighted in the middle box, while those specific to dengue (green box) and COVID-19 (blue box) are shown separately

While dengue was predominantly a childhood infection many years ago, there has been a gradual shift in the age of infection in many countries [63, 83, 115]. Therefore, currently more severe forms of dengue (DHF/DSS) are predominantly seen in the older population in some countries, where CFRs tend to be higher than in younger individuals [79, 83]. However, some studies have shown that CFRs are higher in children, and these differences possibly reflect the differences in disease epidemiology in different countries [119]. Men were significantly more likely to have severe illness in COVID-19, whereas no such associations have been seen with dengue [39, 60]. The presence of metabolic disease, diabetes, hypertension, chronic kidney disease and obesity have shown to be independently associated with the development of more severe illness [55, 72, 102, 110] (Fig. 1). Similarly, mortality rates are higher in elderly individuals and in those with comorbidities when infected with SARS-CoV-2 and influenza. However, in contrast to COVID-19, influenza and many other respiratory infections also cause severe disease in younger children [27, 95]. There are other differences in risk factors for COVID-19 compared to dengue. For instance, while those who were immunosuppressed or those with malignancies were significantly more likely to develop severe COVID-19, whereas such individuals are not at higher risk of severe dengue [39].

COVID-19 or dengue in pregnancy are also associated with a higher risk of severe disease and higher mortality rates [29, 101]. Maternal death was 450 times higher in pregnant women with DHF compared to dengue in non-pregnant females [101] and dengue in pregnancy was associated with a high incidence of acute renal and liver failure, acute respiratory distress and an increased need for ventilatory support [15]. Dengue in pregnancy was associated with a higher incidence in preterm delivery, still birth and low birth weight neonates, similar to COVID-19 in pregnancy, while both infections were not associated with congenital abnormalities of the fetus [100, 124].

Although mechanisms underlying the increased risk of severe dengue and COVID-19 in the elderly, those with comorbidities and in pregnant women are not entirely clear, it could be due to multiple causes such as immunosenescence, an aberrant immune response, pre-existing endothelial dysfunction worsening disease outcome and many other factors [49]. It would be important to further investigate the mechanisms by which these vulnerable groups are more prone to severe illness to provide better preventive methods and treatment modalities.

Interestingly, there has been there are geographical differences in mortality and morbidity due to both dengue and COVID-19. For instance, while the incidence of dengue is similar in many Asian countries and South America, the age standardized death rates and DALYs are significantly lower in South America [139]. Although the reasons for these differences in mortality and morbidity is not known, it could be due to the differences in the DENV that circulate in different regions, vector competence in transmission, force of infection and age of population affected. Similarly, the hospitalizations and mortality rates due to COVID-19 have shown to vary widely between many countries, which could be attributed to differences in COVID-19 vaccination rates, circulating variants, age of population and reporting of COVID-19 deaths [21]. Therefore, although many Asian and African countries have reported lower mortality rates than Europe and Northern America, despite lower vaccination coverage, this is possibly due to inaccurate reporting as many of these countries have reported high levels of excess mortality [21]. However, some countries in Asia (e.g. Sri Lanka) reported lower mortality rates during the omicron wave compared to Europe and North America, with lower excess mortality rates than these countries, despite significantly lower vaccine coverage [21, 46]. Therefore, it would be important to further investigate the reasons for differences in mortality rates in different populations in different geographical regions for both infections.

### Infection characteristics due to dengue and COVID-19

Infections with the DENV occurs following feeding by an infected mosquito of the Aedes species, where the virus infects many innate immune cells and keratinocytes. The time from onset of infection to onset of symptoms (incubation period) for the four DENVs has shown to a mean of 5.9 days [35, 96]. Controlled human challenge models have shown that the duration of viraemia was a mean of 6.8 days, with those who were challenged with a high dose of virus having a longer viraemia than those challenged with a lower dose [35]. Although many different cells have shown to be permissive to infection by the DENV in cell culture [97], autopsy studies have shown that apart from immune cells such as monocytes, dendritic cells, mast cells, the DENV readily infected hepatocytes, kuffer cells, alveolar macrophages, and macrophage like cells in the lymph nodes and spleen [7, 11]. Although some autopsy studies have shown the presence of viral antigens in neurons, kidney cells and endothelial cells, evidence of viral replication within these cells have not been demonstrated [7, 11].

SARS-CoV-2 virus initiates infection by entering cells expressing ACE2, and engagement of the receptor binding domain (RBD) of the virus with ACE2 exposed the cleavage site in S2, which is subsequently cleaved by TMPRSS2 [53]. Following infection of the ciliated cells in the nasal epithelium and type II alveolar cells, the incubation period has shown to be on average 6.38 days, ranging from 2.33 to 17.6 days before patients show symptoms, based on a meta-analysis [34]. However, the incubation period of omicron BA.1 and BA.2 sub-lineages was shown to be shorter than delta and previous variants, which could have contributed to their higher transmissibility [91, 123]. ACE2 is expressed on many cells in addition to the ciliated cells in the nasal epithelium and type II alveolar cells in the lungs, which are initially infected with the virus. Due to the wide expression of ACE2, the SARS-CoV-2 virus has shown to infect the enterocytes, cells in the kidney, heart muscle and testis [53]. The increased susceptibility to severe disease has been attributed to different levels of expression of ACE2 in those with comorbidities [53]. Neuronal cells have been infected in vitro and in animal models, and cerebral atrophy and tissue damage in cortical areas of the brain has been observed in SARS-CoV-2 infected individuals, suggesting that the virus may directly infect the brain [31, 107]. However, similar to the observations in dengue, although endothelium dysfunction plays a significant role in the pathogenesis of COVID-19, there is limited evidence that the virus infects the endothelium in acute SARS-CoV-2 infection [110].

In summary, both the DENV and SARS-CoV-2 infects many type of immune cells and many organs in the body, leading the a widespread infection. While evidence of active replication of the SARS-CoV-2 of the myocardium, kidney, spleen and intestines have been demonstrated, such evidence of active replication within these organs is not seen in dengue, due to the smaller number of studies which has explored this.

### Innate immune responses and cytokine storms in dengue and COVID-19

Although SARS-CoV-2 initiates infection by infecting many different types of cells in the respiratory epithelium, it is known to cause a systemic infection in some individuals infecting the gastrointestinal tract, heart, brain and many other organs [114]. The DENV is also known to infect monocytes, dendritic cells, hepatocytes, keratinocytes and many other cells, while monocytes were shown to be the cell most susceptible to the virus [84]. A dysfunctional immune response by monocytes and other innate immune cells resulting in a delayed interferon response, an increase in proinflammatory cytokines and chemokines such as IL-1-β, TNF-α, CXCL-10, IL-10, IL-18, IL-8 with an increase in many inflammatory lipid mediators is seen in patients who progress to develop severe forms of dengue (DHF) [57, 61, 84]. An impaired and delayed IFN response has shown to associate with a prolonged viraemia and progression to severe disease in COVID-19, and the high levels of IFNs later in the disease were seen to worsen the immunopathology (Fig. 1) [102]. Many DENV proteins inhibit type I IFN production by inhibiting Tyk2 activation and STAT1 phosphorylation, downregulation of STAT2 phosphorylation and by inhibiting STAT2 phosphorylation [17]. In vitro, treatment of HepG2 cells with IFNα and IFNβ prior to infection with the DENV was shown to significantly reduce viral loads in these cells [25]. Therefore, type I interferons appear to play an important role in inhibition of DENV replication and in fact, those who proceeded to develop severe dengue were shown to have reduced levels of plasma IFNα and IFNβ [120]. As seen in dengue, type I IFN responses were shown to be impaired in those who progress to develop severe COVID-19 [72, 102]. Many SARS-CoV-2 structural and non-structural proteins inhibited the type I IFN response by Tyk2 activation, STAT1 and STAT2 phosphorylation and inhibition of IFN signaling [36, 38, 67]. Apart from the SARS-CoV-2 evading immunity by blocking type I IFN production, patients who progress to develop severe COVID-19 have shown to have autoantibodies against IFNα and other type I IFNs [9]. Neutralizing autoantibodies to IFNs, had not been detected in individuals with mild or asymptomatic COVID-19, highlighting the importance of type I IFNs in protection against severe COVID-19[9]. Therefore, an impaired and delayed type I IFN response leads to severe dengue and COVID-19.

**Fig. 2:**
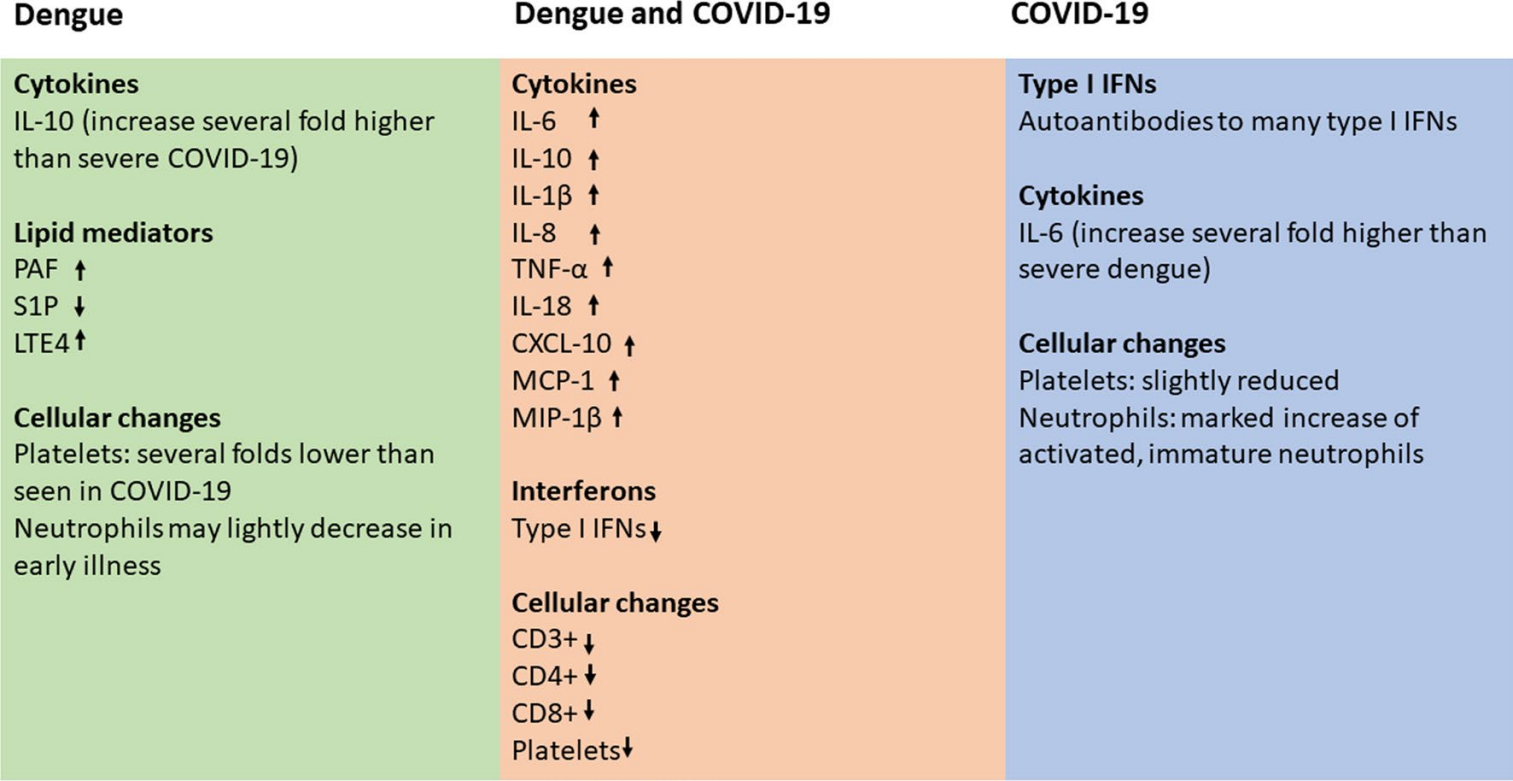
Changes in the innate immune responses, cytokines and chemokines and haematological parameters in patients with severe dengue and COVID-19. The common changes are highlighted in the middle box, while those specific to dengue (green box) and COVID-19 (blue box) are shown separately

Similar to dengue, those who proceed to develop severe COVID-19 have high levels of many proinflammatory cytokines such as IL-6, IL-1β, IL-10, CXCL-10, MCP-1 and the cytokine storm is shown to associate with both severe dengue and COVID-19 (Fig. 2) [137]. Although similar types of cytokines and chemokines are elevated in both dengue and COVID-19, there are many differences in the relative changes of these mediators [23]. For instance, IL-6 levels were shown to correlate with clinical disease severity in COVID-19 and IL-6 receptor antagonists were shown to improve outcomes including survival in critically ill patients [50, 140]. Although IL-6 levels were high in patients with dengue who proceeded to develop DHF, IL-6 levels were several fold lower in the critical phase in patients with dengue compared to COVID-19 (Fig. 1) [23]. High levels of IL-10 levels in early illness in patients with COVID-19 were shown to associate with poorer disease outcomes and was shown to be a predictor of severe disease along with IL-6 [45, 141]. IL-10, which is a potent immunosuppressive cytokine has also shown to act as a proinflammatory cytokine, when elevated with other cytokines [51]. IL-10 is thought to contribute to disease pathogenesis in COVID-19 by either due to its action as an immunostimulatory molecule or because of inability to suppress the hyperinflammation state [51]. However, dengue patients who proceeded to develop DHF had several fold higher levels of IL-10 (mean levels 1331 pg/ml) when compared to those who developed severe COVID-19 (mean 57.3 pg/ml) [23]. In dengue, IL-10 levels in early illness were an important predictor of developing severe disease [23]. IL-10 was shown to suppress DENV specific T cell responses, which could contribute to disease pathogenesis [82].

### Changes in haematological parameters in dengue and COVID-19

The viraemic/febrile phase of dengue is characterized by a leucopenia with a slight decline in platelet counts [127]. Marked leucopenia with a drastic reduction in platelet counts is seen in those who progress to develop DHF, with a rise in the haematocrit due to fluid leakage [127]. Leucopenia is considered a warning sign of development of severe dengue and lymphopenia has shown to correlate with clinical disease severity [54, 127]. The lymphopenia in dengue is predominantly due to reduction in T cells due to apoptosis, although a reduction in B cells was also seen (Fig. 2) [81]. Although the mechanisms of T cell apoptosis are not clear, serum IL-10 correlated with T cell apoptosis, while inversely correlating with T cell numbers [81]. Lymphopenia is also seen in COVID-19 and is shown to correlate with clinical disease severity [20]. While lymphopenia is mainly due to reduction in CD8+ T cells, reduction in all types of lymphocytes (CD4+ T cells, B cells and natural killer cells), is seen in patients with severe COVID-19 (Fig. 2) [20]. In contrast to dengue, severe COVID-19 is associated with a marked increase in neutrophils in the nasopharynx, lung and in blood, which are highly activated and show an immature phenotype [105]. Neutrophils have been shown to contribute to disease pathogenesis in part by release of neutrophil extracellular traps (NET), which was a result of NLRP3 activation [8]. Although an increase in neutrophils is not observed in dengue, NLRP3 activation in many types of cells has been observed with an increase in NET components in the serum of patients with DHF, suggesting that activation of neutrophils is likely to play a role in severe dengue [48, 62, 99].

### Antibody responses in patients with dengue and COVID-19

**Fig. 3:**
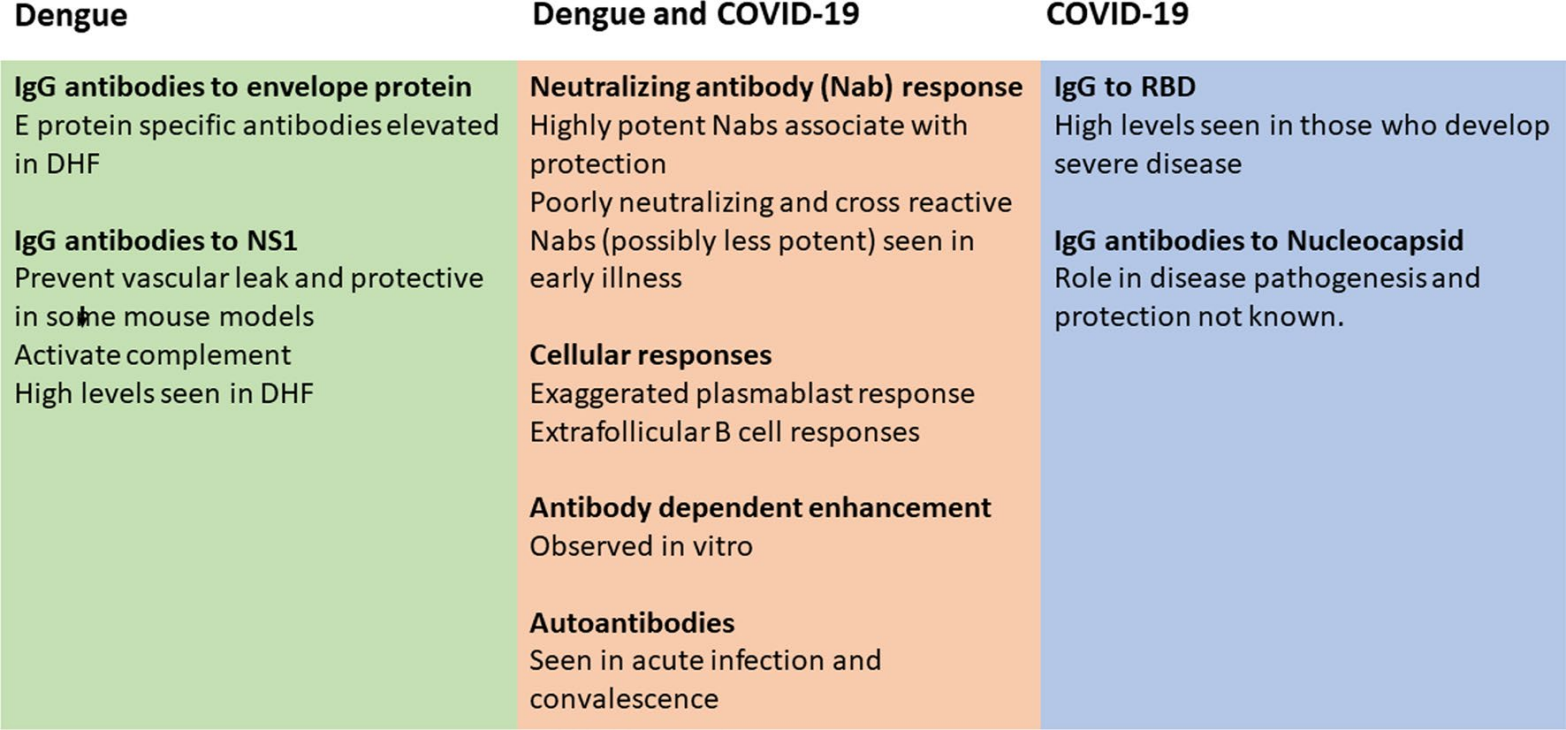
Antibody and B cell responses in patients with severe dengue and severe COVID-19. The common changes are highlighted in the middle box, while those specific to dengue (green box) and COVID-19 (blue box) are shown separately

High levels of neutralizing antibodies (Nabs) following vaccination has shown to prevent infection with the SARS-CoV-2 to a certain degree and associate with protection [66]. DENV serotype specific Nabs have shown to protect against re-infection with the same serotype, while higher levels have also shown to offer protection against symptomatic disease for infection with other serotypes [65]. However, the Nabs antibodies and antibodies directed against the envelope and NS1 protein in dengue can also associate with disease pathogenesis as shown in some studies [24, 55]. The risk of developing DHF is substantially higher in a secondary dengue infection, in which the individual is infected with a different DENV serotype than the earlier infecting serotype [37]. This increase in disease severity is thought to be due to antibody dependent enhancement (ADE), where poorly neutralizing, highly cross-reactive antibodies enhance DENV infection in FcγR-expressing cells [64, 113]. Internalization of these antigen–antibody complexes further leads to disease pathogenesis by inducing IL-10 production by monocytes [118] and DENV-specific afucosylated IgG1 subclass of antibodies further enhanced infection by binding to the activating FcγRIIIA type Fc receptors [122]. Antibodies to NS1, which is a secretory protein of DENV has shown to be protective in some mouse studies [10], while other studies show that NS1 antibody levels are elevated in those who develop DHF [55], and that these contribute to vascular leak by cross-reacting with endothelial cells and inducing apoptosis [75] and by activating complement [6] (Fig. 3). However, as most individuals develop asymptomatic or mild dengue despite the presence of antibodies to the previous DENV serotypes, the type, quality and quantity of DENV specific antibodies that associate with protection is not known.

ADE has been widely discussed in SARS-CoV-2 infection and shown to occur in vitro through multiple mechanisms such as C1q mediated ADE in Vero E6 cells [98], in FcγRIIB expressing B cells [121] and through FcγRIIA and FcγRIIIA receptors in monocytes [80]. However, the clinical significance of ADE in SARS-CoV-2 infection is not known. Although Nabs have shown to be protective in SARS-CoV-2 infection, high levels of virus specific Nabs antibodies and receptor binding domain specific antibodies during early illness have been associated with a worse disease outcome in some studies (Fig. 3) [59, 78, 138]. The extent to which the antibody levels are secondary to high levels of viral replication is not fully resolved. Nabs measured by the surrogate Nabs test (sVNT) showed that in fact, the antibody responses were highest and appeared earlier in those who succumbed to their illness [59]. However, other studies have shown that the presence of highly potent Nabs during early illness was associated with early virus clearance and improved survival [27, 41]. However, unlike in dengue, where infection with a different DENV serotype is a risk factor for severe disease, possibly due to ADE, this has not been seen with SARS-CoV-2 infection. Although there are several different SARS-CoV-2 variants of concern, and many individuals have been infected with a different variant than the one that cause the initial infection, this has not shown to predispose to severe clinical disease [4]. In fact, immune responses generated by natural infection were shown to be longer lasting that those induced by vaccination, and prior natural infection was shown to protect against development of severe clinical disease [4].

A high frequency of activated plasmablasts is seen in patients with severe COVID-19 [12], which is also a feature of severe dengue [40, 132, 136]. Nabs in COVID-19 were shown to be generated by extrafollicular B cells, which correlated with disease severity [135]. Therefore, while high antibody titre may be secondary to high levels of viral replication, it is also possible that antibodies that are generated by an extrafollicular B cell response are less potent and therefore, instead of neutralizing the virus efficiently, they may lead to disease pathogenesis by multiple mechanisms [135]. Extrafollicular B cell responses are also seen in systemic lupus erythematosus and such responses have shown to generate autoreactive antibodies, which is also a feature in COVID-19 [22, 134]. Although the presence of extrafollicular B cell responses have not been studies in dengue infection, poorly neutralizing Nabs have shown to associate with severe disease [24]. Furthermore, a high prevalence of antinuclear antibodies have been detected in the convalescent phase of patients with acute dengue [43]. Therefore, in both COVID-19 and in dengue, while certain types of virus specific antibodies may appear to contribute to disease pathogenesis by many different mechanisms, highly potent Nabs appear to be protective. In order to develop safer and effective vaccines, it would be important to further study the type, quantity and quality of antibody responses that associate with protection, including the type and mechanisms of antibodies that lead to disease pathogenesis.

### T cell responses in dengue and COVID-19

**Fig. 4:**
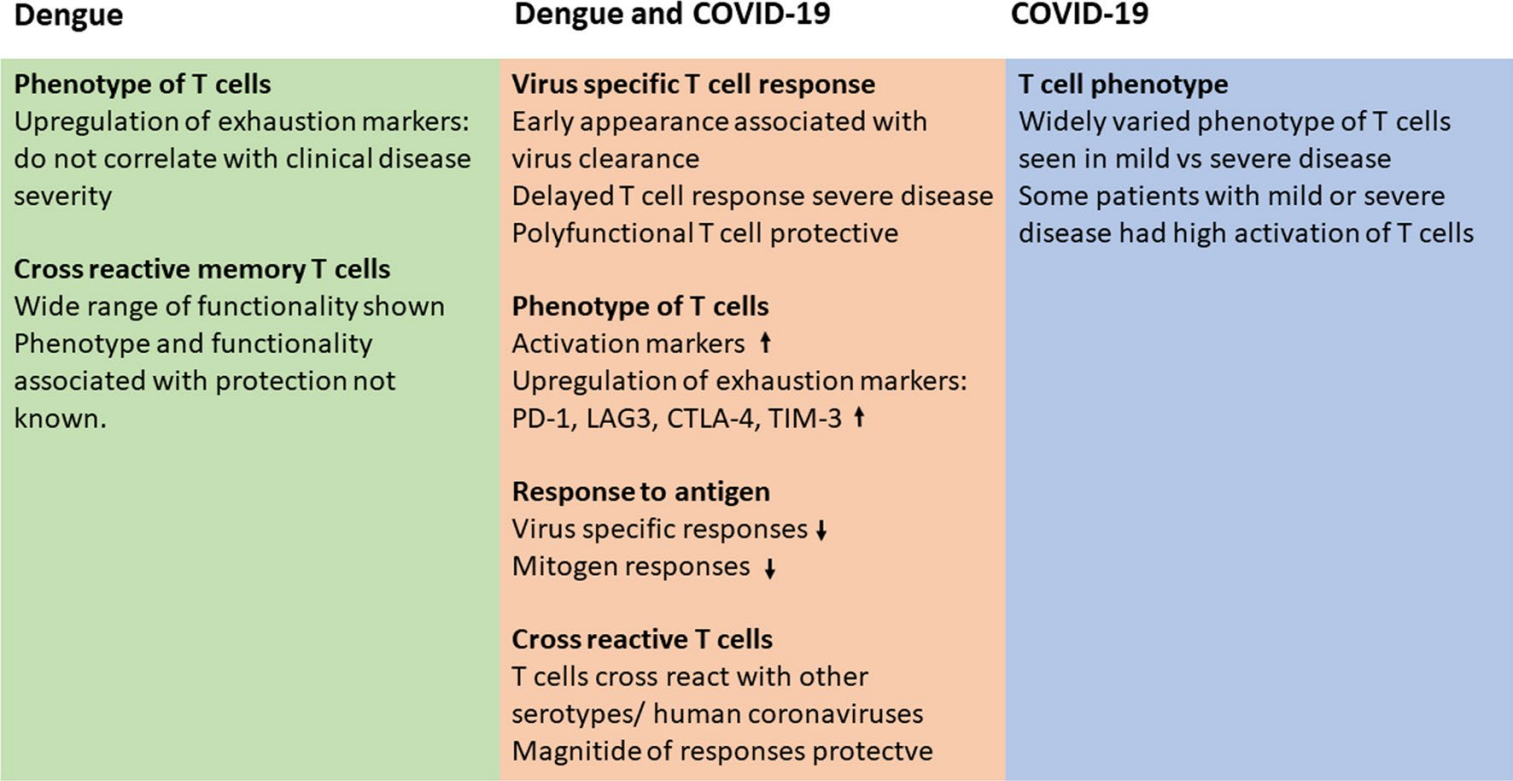
T cell responses in patients with severe dengue and severe COVID-19. The common changes are highlighted in the middle box, while those specific to dengue (green box) and COVID-19 (blue box) are shown separately

For many years DENV specific cross-reactive T cells were thought to be involved in disease pathogenesis [5, 30, 93]. Although patients with more severe forms of dengue had a higher magnitude of cross-reactive T cells, these were only detected during the convalescence period, and were either not detected or were seen in very low frequency in the critical phase [32]. Recent studies have highlighted the importance of DENV specific T cells in reducing disease severity and have shown that early appearance of virus specific T cells correlated with resolution of viraemia and with less severe disease (Fig. 4) [130]. Those with HLA types that were associated with more severe dengue had a lower frequency of DENV specific T cell responses, whereas T cell responses specific for the HLA alleles associated with protection were significantly higher dengue infections [125]. Furthermore, it was shown that the presence of multiple cytokine producing polyfunctional T cells was associated with milder dengue [131].

Acute SARS-CoV-2 infection is associated with a varied magnitude and functionality of the T cell response. Those who had severe disease had a preferential loss of CD8+ T cells compared to CD4+ T cells, a T cell phenotype characterized by activated CD4+ and CD8+ T cells, and CD8+ T cell displaying T cell exhaustion markers such as PD-1, CTLA-4, LAG3 and TIM-3, and a reduced frequency of follicular helper T cells (Fig. 4) [26, 70, 88]. Several negative T cell regulatory molecules such as CTLA-4, LAG3 and PD-1 have shown to be upregulated in patients with dengue [3, 18]. In both infections, CD8+ T cells of those with severe illness had a reduced cytokine production upon stimulation with mitogens and with peptides [18, 82, 142]. As patients with severe COVID-19 had either an unresponsive or suboptimal virus-specific T cells or an exaggerated T cell response, it would be important to understand the T cell responses that associate with protection for better vaccine design.

SARS-CoV-2 virus specific cross-reactive T cells have shown to be present in unexposed donors and are thought to be due to the presence of cross-reactive T cell responses for other seasonal human coronaviruses [87]. In SARS-CoV-2 infection, the presence of IL-2 producing cross-reactive T cells was shown to be protective in house-hold contacts and was associated with a negative PCR [68]. Furthermore, the presence of these immunodominant CD4+ T cell epitopes that cross react with SARS-CoV-2 and other human coronaviruses have shown to decline with age, which has been suggested as a contributory factor for severe disease in the elderly [77]. Although omicron and its emerging sub-lineages completely escaped antibody mediated immunity induced by two doses of many of the COVID-19 vaccines, they were still shown to be protected by severe disease and hospitalization due to the presence of robust T cell responses [94]. Previous infection and many of the COVID-19 vaccines, were shown to induce a high magnitude of T cell responses with a broad recognition of T cell epitopes of many of the viral proteins [94]. T cells have shown to play an important role in viral clearance especially in the context of low antibody levels in non-human primates [89]. Therefore, although the emergence of sub-lineages of omicron such as BA.4 and BA.5 are shown to be more immune evasive than all existing variants, as a result of further mutations in the Nab binding sites. However, due to the breadth of the T cell response induced following natural infection and vaccination, infection with these variants is likely to associate with a relative reduction in clinical disease severity, despite infection, as evidenced with the global decline in COVID-19 mortality rates [16].

Although many studies have been carried out in understanding the T cell responses in SARS-CoV-2 infection, the significance of DENV-specific cross-reactive memory T cell responses subsequent clinical disease severity when infected with the DENV has not been extensively studied. However, those with varying severity of past dengue, were shown to have varied frequencies of T cells and T cell functionality [56], possibly related to the timing of the previous infection and the number of previous DENV infections.

### Endothelial dysfunction in dengue and COVID-19

Endothelial dysfunction leading to vascular leak in the hallmark of DHF, which occurs due to viral factors, cytokines and inflammatory mediators [85]. The vascular leak phase which lasts for 24 to 48 h in dengue, occurs around 3 to 5 days since the onset of illness, and this leads to pleural effusion, ascites and shock [85]. The dengue NS1 protein, which is a secretory protein has shown to contribute to endothelial dysfunction by disrupting the endothelial glycocalyx layer [104]. Many inflammatory lipid mediators such as platelet activating factor (PAF), vascular endothelial growth factor (VEGF), angiopoietin-2 (Ang-2) has shown to cause endothelial dysfunction and phospholipase A2 enzymes that generate PAF were shown to be elevated during early illness in dengue [57, 58, 85]. Dengue NS1 was also shown to induce phospholipase A2 enzymes, inflammatory cytokines and prostaglandins, further contributing to endothelial dysfunction [112].

Endothelial dysfunction is also a feature of severe COVID-19 with high levels of VEGF, Ang-2, osteopontin, although the vascular leak is not as prominent as seen in dengue [106]. In comparison to those who died of acute respiratory distress syndrome (ARDS) in influenza infection, those who died of COVID-19 had severe endothelial injury with membrane disruption along with the presence of virus within the endothelial cells [1, 14]. Autopsy studies have shown the presence of venous and arterial platelet–fibrin microthrombi in many organs in many patients with COVID-19, which is thought to occur due to endothelial activation [1]. Many factors are thought to contribute to the endothelial dysfunction and occurrence of a prothrombotic state, such as direct infection of the endothelium with SARS-CoV-2 causing endothelial damage, inflammatory cytokines such as IL-6, hyperplasia of the endothelium due to lung tissue ischaemia and activation of neutrophils and monocytes along with platelets facilitating microthrombi formation [14, 106].

The coagulopathy in COVID-19 is associated with high levels of d-dimer, fibrinogen and von Willebrand factor, with modest reductions in platelet counts and slightly prolonged or normal prothrombin and activated partial thromboplastin times (APPT) [14, 116]. In contrast to COVID-19, dengue is associated with a bleeding tendency, with marked reductions in platelet counts, with prolonged prothrombin and APPT in patients with severe disease [2, 127]. However, those with DSS and severe COVID-19 had elevated levels of thrombomodulin, plasminogen activator inhibitor type 1 and von Willebrand factor antigen suggesting that like COVID-19, activation of procoagulant mechanisms also occur in severe dengue [28, 44, 106, 133]. Therefore, although the extent of endothelial dysfunction in both infections is a marker of clinical disease severity, the pathogenesis of endothelial dysfunction and the coagulation disturbances that occur as a result of this appear to be different.

### Long term sequel of dengue and COVID-19

Many individuals report fatigue following dengue infections with 32% reporting fatigue at 2 months post-infection [111]. A smaller study showed that approximately 50% of individuals who had symptomatic dengue have persistent symptoms such as muscle and joint pain, headache and insomnia, 2 years post-infection, although there was no control group included in this study [42]. The persistence of symptoms was associated with polymorphisms of the FcγRIIa gene, presence of anti-nuclear antibodies and immune complexes [42]. An increased incidence of several different types of autoimmune disease such as Reiter’s syndrome, myasthenia gravis, autoimmune encephalomyelitis and systemic vasculitis have been reported following dengue [73]. In Mexico, the annual burden due to persistent symptoms following dengue has shown to cost US$ 22.6 million annually [117]. However, the proportion of individuals who develop chronic fatigue and the pathogenesis of these long-term complications in dengue has not been studied.

The long-term sequelae of COVID-19 is well recognized and the term ‘long COVID (post-acute sequel of COVID-19)’ is commonly used to describe the symptoms that occur following COVID-19. Although long COVID is more frequent following severe illness, it has also been reported in those with mild illness with chronic fatigue, persistent lung symptoms, olfactory symptoms, neurological, gastrointestinal symptoms and endocrine abnormalities being described, lasting for months post-infection [13, 92]. 52% of young adults with mild illness (home isolated) reported symptoms of fatigue, dyspnoea, cognitive dysfunctions and loss of taste and smell, at 6 months post-infection [13]. Abnormal lung function with fibrosis and structural changes, myocarditis, thromboembolism, chronic kidney disease, skin disease and structural changes in the brain [31, 92, 109]. Although the pathogenesis of long COVID appears to be multifactorial, persistence of the virus, alteration of immune homeostasis leading to persistent activation of the immune system, alternation of immunometabolic pathways and autoimmunity have been proposed as possible mechanisms [92, 103]. Although myocarditis (11.3%) [74], acute liver failure (0.31%) [71] and acute renal failure (2 to 5% of patients with severe dengue) [76] is reported to occur in dengue, long term organ dysfunction has not been reported in those who recover. Therefore, although chronic fatigue, the presence of certain autoantibodies and an increased risk of autoimmune diseases have been reported following dengue, dengue does not appear to associate with the occurrence of severe disabling and serious long-term sequelae seen following SARS-CoV-2 infection.

## Conclusions

The concurrent occurrence of dengue and COVID-19 outbreaks in many Asian and Latin American countries are likely to cause a significant burden to the health care systems of these resource poor countries. Since the initial clinical presentations of these two infections are quite similar, it would be a challenge to clinically differentiate these two infections. In addition, there are many similarities in the immunopathogenesis between dengue and SARS-CoV-2 infection with a dysfunctional immune response leading to a cytokine storm and immunopathogenesis, delayed virus specific T cell responses failing to clear the virus, extra follicular B cell responses and exaggerated plasmablast responses associating with severe disease and endothelial dysfunction. However, while dengue is usually associated with a tendency to bleed, development of micro and macrothrombi is a hallmark of severe COVID-19. Apart from the differences in coagulation abnormalities and the differences in the cytokine storms and mediators that cause endothelial dysfunction, there are also many differences especially in long-term disease sequelae. Although both infections occur due to very different routes (respiratory infection vs mosquito borne infection), it would be important to study the parallels between the immunopathogenesis of both infections for development of more effective vaccines and to develop therapeutic interventions.

## Data Availability

All data is available in the manuscript and figures.

## Abbreviations

DENV: Dengue virus
DF: Dengue fever
DHF: Dengue haemorrhagic fever
DSS: Dengue shock syndrome
CFRs: Case fatality rates
APPT: Activated partial thromboplastin times
IFN: Interferon
NET: Neutrophil extracellular traps
ADE: Antibody dependent enhancement
sVNT: Surrogate virus neutralizing test
VEGF: Vascular endothelial growth factor
Ang-2: Angiopoietin-2
